# Primary pulmonary extranodal natural killer/T-cell lymphoma (ENKTL), nasal type: Two case reports and literature review

**DOI:** 10.1097/MD.0000000000020822

**Published:** 2020-06-26

**Authors:** Qun Hu, Liyu Xu, Xiaoming Zhang, Jie Wang, Zizi Zhou

**Affiliations:** a Department of Respiratory, Critical Care and Sleep Medicine, Xiang’an Hospital, Xiamen University, Xiamen; b Department of Pulmonary and Critical Care Medicine, Fuzhou First Hospital, Fujian Medical University, Fuzhou; c Department of Cardio-Thoracic Surgery, Shenzhen University General Hospital; d College of Pharmacy, Shenzhen University, Shenzhen, China; e Department of Plastic and Reconstructive Surgery, BG Unfallklinik Ludwigshafen, University of Heidelberg, Heidelberg, Germany.

**Keywords:** case reports, extranodal natural killer/T-cell lymphoma, literature review, nasal type, primary pulmonary lymphoma

## Abstract

**Introduction:**

Extranodal natural killer/T-cell lymphoma (ENKTL) – nasal type is an aggressive form of malignant non-Hodgkin lymphoma with a very poor prognosis. Especially primary pulmonary ENKTL is a relatively rare form of non-Hodgkin lymphoma. Until now, the prevalence of primary pulmonary ENKTL is unknown. Since 2001, only 18 cases of primary pulmonary ENKTL have been published, in addition to the 2 cases reported here.

**Patient concerns:**

We describe 2 cases of primary pulmonary ENKTL. Both patients were male non-smokers, aged 61 and 49 years. Their main clinical symptoms included cold-like symptoms and intermittent fever (39.3°C and 38.8°C) for some days (40 days and 3 weeks). Both patients had no relevant personal or family medical history.

**Diagnosis:**

The patients were initially misdiagnosed with community-acquired pneumonia. Primary pulmonary ENKTL was confirmed by immunohistochemical staining of computed tomography-guided transthoracic needle biopsy specimens. Both cases were positive for CD56, CD3, and in situ hybridization for Epstein-Barr virus-encoded small RNA, but negative for CD20.

**Interventions:**

Initially, both patients were treated inadequately with intravenous moxifloxacin administration (unknown dosage and 400 mg q.d) in their local hospitals. Once diagnosed with primary pulmonary ENKTL in our hospital, they received 3 cycles of chemotherapy with combined regimens of dexamethasone, methotrexate, ifosfamide, L-asparaginase, and etoposide (SMILE), and in the second patient, bone marrow transplantation was performed following the third chemotherapy cycle.

**Outcomes:**

Clinical follow-up after the chemotherapy showed that the condition of the first patient progressively deteriorated. He died 2 months following the initial diagnosis. However, the presence of the hemophagocytic lymphohistocytosis gradually improved in the second patient during chemotherapy. Ultimately, the second patient died of acute transplant rejection 6 months after the initial diagnosis.

**Conclusion:**

The diagnosis of ENKTL should be considered when patients present with fever and expansile consolidation of the lung not responding to antibiotics. The diagnosis depends on histopathology and immunophenotyping. Percutaneous transthoracic needle biopsy is a safe and effective biopsy method. Chemotherapy may improve the prognosis, but this should be confirmed by prospective multicenter studies.

## 1 Introduction

In 2001, NK/T-cell lymphoma was uniformly defined as an extranodal natural killer/T-cell lymphoma – nasal type (ENKTL) by the World Health Organization.^[1]^ This type of tumor is predominantly located in the upper aerodigestive tract (76%–86%), including the nasal cavity, nasopharynx, paranasal sinuses, and palate.^[2]^ However, primary pulmonary ENKTL cases are relatively rare and only 18 cases have been reported publicly. Primary pulmonary ENKTL is diagnosed based on the following criteria:

1. Neoplastic cells initially appear in lung tissue without other anatomic sites involved;
2. Previous ENKTL history is excluded.^[3]^

We report 2 cases of primary pulmonary ENKTL presenting as pneumonia and review previous cases.

## 2 Case presentations

### 2.1 Case 1

**Figure 1:**
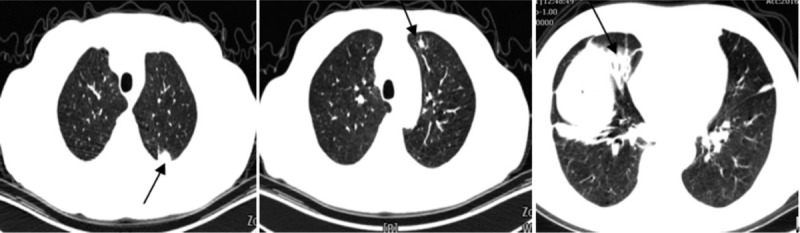
Chest computed tomography showing multiple nodules in the bilateral upper lobes, alveolar infiltration in the left lower lobe, and the absence of lymphadenopathy.

**Figure 2:**
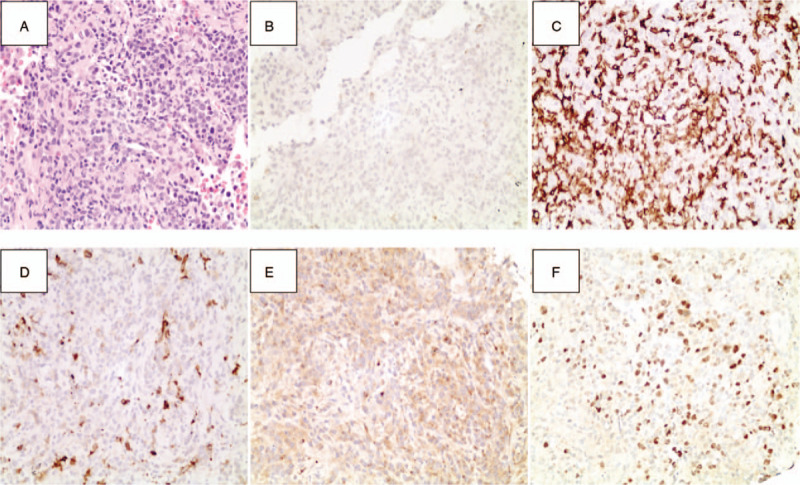
Pathologic findings in CT-guided transthoracic needle biopsy specimens. (A) Histologically, a small number of atypical cells presented with large areas of observable necrosis (HE staining; magnification,×200). (B) Immunohistochemical staining positive for the expression of CD56 (magnification, ×200). (C) Immunohistochemical staining positive for CD3 expression (magnification, ×200). (D) Immunohistochemical staining negative for the expression of CD20 (magnification, ×200). (E) Immunohistochemical staining positive for the presence of granzyme B (magnification, ×200). (F) In situ hybridization positive for EBV-encoded RNA (magnification, ×200).

A 74-year-old man who had never smoked was referred to the Department of Pulmonary and Critical Care Medicine at Fuzhou First Hospital, Fujian Medical University (Fuzhou, Fujian, China) on June 16, 2016, with chief complaints that included fever and cough over 40 days. Initially, the patient had intermittent fever of ≤39.3°C. He received antibiotic treatment (moxifloxacin, unknown dosage) for more than 2 weeks. However, his clinical condition did not improve. The patient was therefore admitted to our hospital for further evaluation and treatment. There was no relevant personal or family medical history for this patient. On admission, his physical examination demonstrated no abnormalities with the notable exception of a body temperature of 38.6°C. The hemoglobin was 10.7 g/dl, the leukocyte count was 440/μl, and the platelet count was 102 × 10^9^/L. The peripheral blood analysis revealed no atypical lymphocytes. The C-reactive protein concentration was 76 mg/L (normal range, 0–8 mg/L), the procalcitonin level was 0.2 ng/ml (normal range <0.05 ng/ml), and the lactate dehydrogenase (LDH) was 542 U/L (normal range 109–245 U/L). The patient was HIV negative. Chest computed tomography (CT) revealed multiple nodules in the bilateral upper lobes of the lung and alveolar infiltration in the left lower lobe, without lymphadenopathy (Fig. 1). A CT-guided transthoracic needle biopsy of the left upper lung was undertaken; the surgical specimen was fixed in 4% formalin, embedded in paraffin, and stained with hematoxylin and eosin (HE). Histologically, a small number of atypical cells were present with large areas of tissue necrosis. Immunohistochemical staining yielded positive results for CD56, CD3, TIA-1, and granzyme B. Furthermore, in situ hybridization for Epstein-Barr virus (EBV)-encoded RNA (EBER) was positive, and the Ki-67 proliferation index was 80% (Fig. 2). However, the atypical cells were negative for CD20, CD5, thyroid transcription factor 1, and synaptophysin. Head and abdominal magnetic resonance imaging, emission computed tomography, bone marrow biopsy, and nasopharyngoscopy were also negative. Based on the morphological and immunophenotypic characteristics, EBER positivity, and lack of extrapulmonary site involvement, the patient was finally diagnosed with primary pulmonary ENKTL. He subsequently received 3 cycles of chemotherapy with combined regimens of dexamethasone, methotrexate, ifosfamide, L-asparaginase, and etoposide (SMILE)^[4]^; however, his condition progressively deteriorated, and he died 2 months following the initial diagnosis.

### 2.2 Case 2

**Figure 3:**
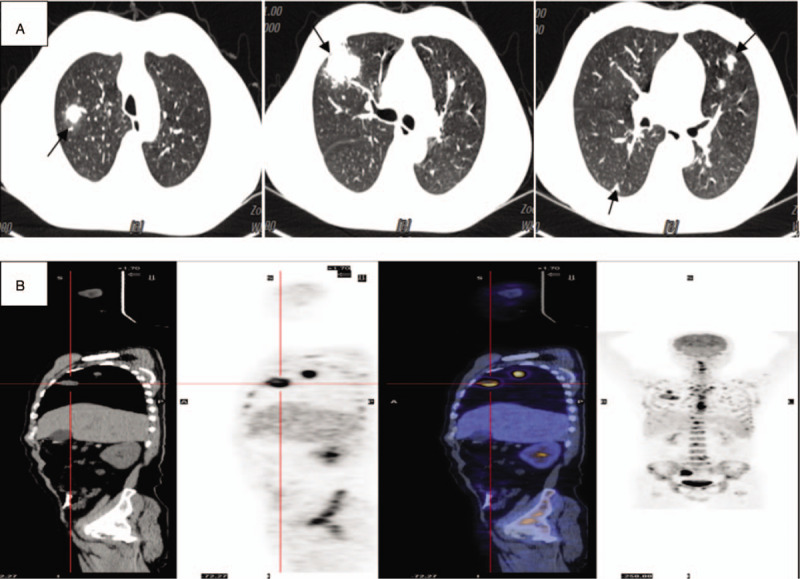
Chest CT scan showing multiple nodes in both lungs. (B) PET imaging revealing lobulated heterogeneous hypermetabolic masses in both lungs, as well as in the pelvic cavity, spleen, and bones. Additionally, multiple lymph node metastases involving the mediastinum and right hilum can be identified.

A 49-year-old non-smoking man was admitted to our hospital on March 28, 2017, with chief complaints of fever and a cold for 3 weeks. At first, the patient had intermittent fever of ≤38.8°C. He was subsequently admitted to a local hospital. Laboratory examinations revealed that the white blood cell count was 6.86 × 10^9^/L, the platelet count was 223 × 10^9^/L, the C-reactive protein was 178 mg/L (normal, 0–8), the procalcitonin level was 0.5 ng/ml (normal < 0.05), the alanine aminotransferase concentration was 170 IU/L, and the LDH was 313 U/L. Chest CT scanning showed multiple massive infiltrates in both lungs. He was then diagnosed with community-acquired pneumonia and treated with moxifloxacin (400 mg q.d., intravenously) for more than a week, although his clinical condition subsequently deteriorated. The patient was, thus, referred to our hospital for further evaluation and treatment. The patient did not present a relevant personal or family medical history. On admission, his vital signs showed a blood pressure of 148/64 mm Hg, a resting pulse rate of 146 beats/minute, a respiratory rate of 21 breaths/minute, and a body temperature of 40.2°C. On physical examination, his face had an acutely ill-looking appearance. He had no peripheral lymphadenopathy, organomegaly, or evidence of skin lesions. Laboratory findings showed a white blood cell count of 1.8 × 10^9^/L, a hemoglobin concentration of 7.3 g/dl, a platelet count of 42 × 10^9^/L, an alanine aminotransferase concentration of 176 IU/L, an LDH level of 313 U/L, and a serum ferritin concentration of > 1,888 ng/ml. In addition, his plasma EBV DNA level was 50,400 copies/ml. Chest CT scanning showed multiple nodes in both lungs (Fig. 3A). Moreover, 18-fluoro-deoxyglucose positron emission tomography (PET) revealed lobulated heterogeneous hypermetabolic masses in both lung fields, as well as pelvic cavity, splenic, bone tissue, and multiple lymph node metastases that involved the mediastinum and right hilum (Fig. 3B). A CT-guided transthoracic needle biopsy of the right upper lung was performed, following which the surgical specimen was fixed in 4% formalin, embedded in paraffin, and stained with HE. Histologically, a small number of atypical cells presented with an inflammatory response and necrosis. Immunohistochemical staining yielded positive results for CD56, CD3, CD2, CD7, granzyme B, and EBER, and the Ki-67 proliferation index was 70%; however, results were negative for CD79a, CK7, CD20, and T-cell-restricted intracellular antigen-1 (TTF-1). The nasopharyngoscopy was negative. Bone marrow aspiration was performed due to cytopenia demonstrating phagocytosis of erythrocytes and platelets. Hemophagocytic lymphohistocytosis was confirmed by cytopenia, fever, splenomegaly, hyperferritinemia, and the presence of hemophagocytosis in the bone marrow. Based on these morphological, immunophenotypic, and cytogenetic findings, in addition to EBER expression in the tumor cells, the patient was finally diagnosed with pulmonary ENKTL. Besides, the PET imaging results did not indicate any involvement of the upper aerodigestive tract. Therefore, it is reasonable to postulate that the lung was the primary site of manifestation in this case. Thus, this patient was treated according to the SMILE chemotherapeutic regimen, and the hemophagocytic lymphohistocytosis gradually improved during chemotherapy. Bone marrow transplantation was performed performed following the third chemotherapy cycle. However, this patient ultimately died due to acute transplant rejection.

## 3 Discussion

### 3.1 Epidemiology

ENKTL is the second most common form of non-Hodgkin lymphoma and accounts for 57.8% of mature T-cell and NK cell lymphomas^[5]^ that are most often seen in Asia and South America whereas being rare in the United States and Europe.^[2]^ This tumor can express both T-cell differentiation antigens and NK cell-associated antigens with unique neoplastic, morphological, immunophenotypic, and biological features and behavior. In addition, EBV plays an important role in the pathogenesis of ENKTL.^[6]^ ENKTL can originate in nasal or extranasal sites but extranasal ENKTL, and especially pulmonary ENKTL, is a relatively rare form of non-Hodgkin lymphoma. Laohaburanakit et al describe that primary pulmonary non-Hodgkin lymphoma is found in only 3% to 4% of cases.^[7]^ To date, the instance of primary pulmonary ENKTL is unknown. Since 2001, only 18 cases of primary pulmonary ENKTL have been reported^[2,3,7–17]^ in addition to the 2 cases presented above. Thus, 20 patients were studied, and their clinical characteristics are summarized in Table 1.

**Table 1:**
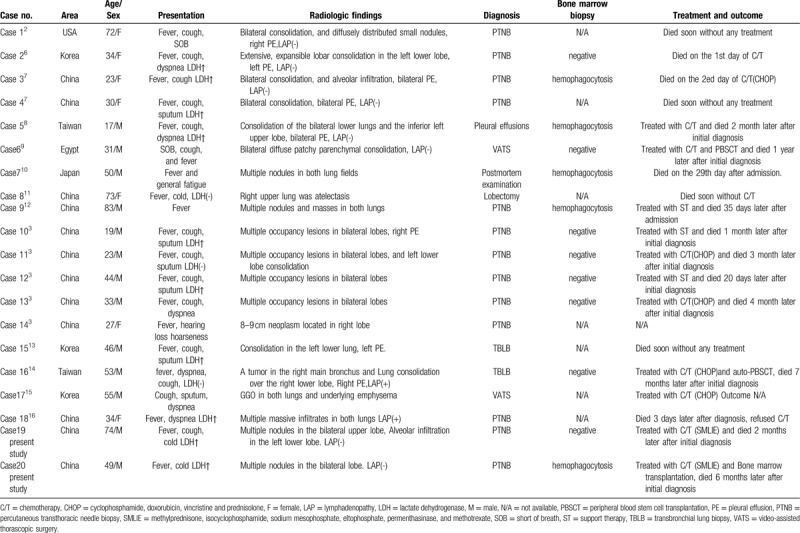
Summary of reported cases of primary pulmonary ENKTL during 2001 and 2018.

### 3.2 Clinical features

The majority of patients with primary pulmonary ENKTL came from Asia, especially from China; only 1 patient was residing in the United States, whereas none was reported from Europe. The patients included 13 males and 7 females with an age range of 17 to 83 years (mean age, 45 years). Most of the patients presented with fever, cough, sputum, and dyspnea. The radiological findings showed consolidation (cases 1–4, 5, 7, 15, and 16), single or multiple nodules (cases 7, 8–14, 19, and 20), alveolar infiltration (cases 18 and 19), atelectasis (case 8), and ground-glass opacification (case 17). Mediastinal lymphadenopathy (cases 16 and 18) and pleural effusion (cases 1–5, 10, 15, and 16) were also reported. These radiographic features should be differentiated from tuberculosis, pneumomycosis, and lung cancer. Usually, chest CT findings of a primary pulmonary NK/T-cell lymphoma vary and are nonspecific. They can be divided into 3 types – nodular or mass-like, mesenchyma-like, and pneumonia-like.^[18,19]^ Thus, it is difficult to differentiate primary pulmonary NK/T-cell lymphoma from pneumonia if the CT of the chest shows pneumonia-like features. However, air bronchograms and halo signs can be regarded useful evidence, because they are present in most primary pulmonary extranodal NK/T-cell lymphoma.^[18,20]^ Moreover, bleeding can be observed surrounding the halo signs. Lymphoma cells can invade blood vessels leading to bleeding into the surrounding tissue. Pathological and immunohistological findings confirm the definitive diagnosis of primary pulmonary extranodal NK/T-cell lymphoma, nasal type (WHO classification).

### 3.3 Histological and immunohistochemical features

The diagnosis of NK/T-cell lymphoma depends on histopathology and immunophenotyping. Lung tissue samples were predominantly obtained by transbronchial lung biopsy, percutaneous transthoracic needle biopsy (PTNB), or surgical lung biopsy. In our literature review, the diagnosis of case 6 was based on pleural effusion cells. Transbronchial lung biopsy was performed in 6 cases, while case 1, and cases 6, 16, and 7 were non-diagnostic, and finally diagnosed by PTNB, surgical lung biopsy, and post-mortem examination, respectively. PTNB was used in 13 of the 20 patients, which might be related to the location of the lesions in the peripheral areas. No complications of pneumothorax and hemorrhage were reported, and thus PTNB was considered a safe and effective method for obtaining a tissue biopsy. A PTNB should also be considered when the clinical symptoms show evidence of deterioration despite adequate antibiotic therapy.

The histological features are shown in Table 2. Almost all cases showed evidence of diffuse infiltration of heteromorphic lymphocytes. Seven cases presented with vascular infiltration, 12 cases with necrosis, and 8 cases with evidence of inflammation. We found it challenging to differentiate ENKTL from other inflammatory disorders, tuberculosis, aggressive NK/T-cell leukemia (ANKL), lymphomatous granulomatosis, and lymphoepithelioma-like carcinoma. Thus, routine immunohistochemical staining is needed. The immunophenotyping of ENKTL can be divided into 4 subgroups: NK cell-related antigen phenotype (CD56); T-cell anti-prototype (CD2, cytoplasmic CD3); cytotoxic antigen phenotype (TIA-1, granzyme B, perforin); and EBV-related antigen phenotype (EBER). Moreover, CD56 is a sensitive biomarker for diagnosing ENKTL; however, the positive rate of this marker is restricted to approximately 82%.^[21]^ Besides, 3 cases in this study were negative for CD56, and the diagnosis for these cases was based on the results from the remaining 3 positive characteristics.

**Table 2:**
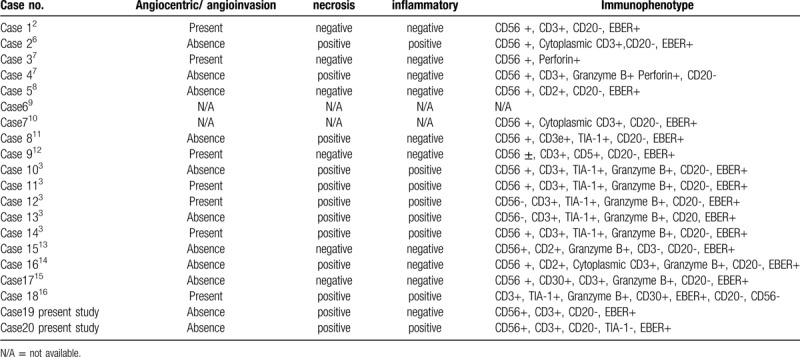
The brief histopathologic findings of primary pulmonary ENKTL.

### 3.4 Treatment and prognosis

The treatment methods for pulmonary ENKTL may include chemotherapy, combinations of radiotherapy and chemotherapy, and hematopoietic stem cell transplantation. However, there is still no strongly recommended treatment option for this condition at present.^[7]^ That said, chemotherapy remains the preferred option for advanced stage and primary extranasal ENKTL,^[12]^ while the 5-year overall survival rate is only 9.0% to 15.6%.^[22]^ In the reviewed literature, 8 patients received chemotherapy, and 1 of them underwent peripheral blood stem cell transplantation. The survival time of these patients ranged between 1 day to 1 year (median survival, 3.5 months) after the initial diagnosis, which is longer than the survival time of patients who refused chemotherapy or received only supportive therapy (median survival, <1 month). Although some cases potentially benefited from chemotherapy or stem cell transplantation, none of these cases experienced a survival of 5 years. ENKTL can easily develop into hemophagocytic lymphohistocytosis, a syndrome with a high mortality rate. In this study, bone marrow biopsies were obtained in 13 patients, and 5 of these 13 patients (38%) developed hemophagocytic lymphohistocytosis. There were no differences regarding age, sex, and prognosis between cases that were negative or positive for hemophagocytosis. This observation might be associated with the high mortality rate seen in ENKTL and the effectiveness of the chemotherapy.

## 4 Conclusion

In conclusion, primary pulmonary ENKTL is an extremely rare disease with a dismal prognosis. It is liable to be misdiagnosed as an inflammatory disorder. Furthermore, the diagnosis of ENKTL should be considered when patients present with fever and expansile consolidation of the lung not responsive to antibiotics. The diagnosis depends on histopathology and immunophenotyping. Furthermore, we found that PTNB is a safe and effective biopsy method. Chemotherapy may improve the prognosis, but this should be confirmed by prospective multicenter studies.

## Author contributions

QH, LX, and ZZ contributed to the study concept, design, as well as data collection and interpretation. QH and ZZ wrote the manuscript. XZ and JW reviewed and edited the manuscript. ZZ was in charge of revising the article and of the final approval of the manuscript prior to submission. All authors read and approved the final manuscript.
